# Developing a Core Outcome Set for Pediatric and Adult Acute and Chronic Pain Extended Reality Trials: Delphi Consensus-Building Process

**DOI:** 10.2196/58947

**Published:** 2025-05-23

**Authors:** Courtney W Hess, Deirdre E Logan, Brittany N Rosenbloom, Giulia Mesaroli, Laura E Simons, Carley Ouellette, Cynthia Nguyen, Fahad Alam, Jennifer N Stinson

**Affiliations:** 1 Department of Anesthesiology, Perioperative, and Pain Medicine Stanford University School of Medicine Palo Alto, CA United States; 2 Department of Anesthesia, Perioperative and Pain Medicine Boston Children's Hospital Boston, MA United States; 3 Department of Psychiatry Harvard Medical School Boston, MA United States; 4 Toronto Academic Pain Medicine Institute Women's College Hospital Toronto, ON Canada; 5 Department of Anesthesiology & Pain Medicine University of Toronto Toronto, ON Canada; 6 Research Institute Hospital for Sick Children Toronto, ON Canada; 7 Institute of Health Policy, Management and Evaluation University of Toronto Toronto, ON Canada; 8 School of Nursing McMaster University Hamilton, ON Canada; 9 Sunnybrook Health Sciences Centre Toronto, ON Canada; 10 Lawrence Bloomberg Faculty of Nursing University of Toronto Toronto, ON Canada

**Keywords:** pediatric, adults, acute pain chronic pain, extended reality, virtual reality, augmented reality, core outcome set, interventions, clinical trials

## Abstract

**Background:**

Appropriate outcome assessment strategies and high-quality trials are critical to advancing care of patients with acute and chronic pain. Using extended reality (XR), namely, virtual and augmented reality, as a nonpharmacological treatment for pain has accelerated in the last decade. XR allows users to engage completely in immersive, gamified, sensorial digital experiences. Currently, no standardized approach to assessing outcomes of XR-based interventions for pain exists.

**Objective:**

Our aim was to recommend a core set of outcomes for pediatric and adult acute and chronic pain XR intervention trials.

**Methods:**

To identify core outcomes, we conducted a multiphase process. In phase 1, we conducted systematic reviews on XR in pediatric and adult acute and chronic pain trials to identify the most common core outcome domains assessed in existing published studies. Primary outcome domains were identified and informed the development of the survey for phase 2, a Delphi survey of clinicians and researchers who were actively researching or using XR for pain treatment. Together, results from the systematic reviews and Delphi survey responses were collated, and in phase 3, a 2-day in-person meeting was held to reach consensus on recommended outcome domains for adult and pediatric acute and chronic pain XR clinical trials. This was followed by 2 additional rounds of the Delphi survey to broaden consensus and refine the domains and definitions. Following the Outcome Measures in Rheumatology guidelines for consensus building, outcomes were organized into 3 categories: mandatory, important to consider but optional, and research agenda.

**Results:**

A systematic review including XR trials for adult and pediatric acute and chronic pain was conducted in March 2023, and 90 pediatric and 104 adult studies were included. The round 1 Delphi survey, completed by 66 respondents, revealed the following commonly measured outcomes: pain intensity or quality, distraction, anxiety or fear, satisfaction, and adverse events. Respondents indicated the following domains to be of highest importance to measure in studies: safety, feasibility, and acceptability; pain intensity or quality; pain interference or functioning; emotional functioning; and user experience or immersion. By unanimous vote at the consensus conference, pain severity, adverse events, user experience, and psychological constructs were identified as mandatory domains to be assessed in all XR trials for acute and chronic pain, with the addition of pain interference for chronic pain trials. Physiological markers and physical function were deemed important-to-consider but optional domains. Additional emerging areas for future research did not obtain sufficient support in the consensus process but were noted.

**Conclusions:**

An established core outcome set will help strengthen the emerging evidence base supporting XR interventions for children and adults with pain. Future work is underway to provide recommendations for appropriate validated measures to assess each established outcome domain.

## Introduction

### Background

Acute pain (eg, injury, procedural pain, and medical treatments), recurrent pain (eg, headaches), and chronic pain (eg, musculoskeletal and complex regional pain syndrome) are common among adults and children and can have negative short- and long-term physical and mental health consequences. Poorly treated acute pain can transition to chronic pain, which affects 1 in 5 people [1-3] and has negative impacts on all aspects of quality of life [4], as well as high economic costs to families and society [5,6]. Chronic pain can also lead to a higher risk for opioid misuse [7] and high health care use [8,9]. Optimal pain treatment uses a multimodal (3P) approach that includes pharmacological, physical, and psychological strategies. Behavioral health treatments are particularly attractive to mitigate risks of opioid misuse. Biopsychosocial pain treatments aim to reduce pain intensity and pain-related disability by improving the person’s emotional, social, vocational, or physical functioning [10]. However, engagement with biopsychosocial treatments can be difficult due to fear and avoidance of pain [10], requiring innovative solutions.

To mitigate fear of pain and increase engagement in health care treatments, integration of extended reality (XR), namely, virtual reality (VR) and augmented reality (AR), has accelerated in the last decade [11]. XR interventions aim to engage a user in a simulated environment that they perceive to be real. VR transports the user to an alternative environment, whereas AR enhances an existing physical environment with additional virtual objects embedded in that real setting [11-14]. XR allows users to engage completely in immersive, gamified, and sensorial digital experiences. Experts have postulated several mechanisms through which XR can reduce pain or pain-related disability, including cognitive (eg, distraction from pain), emotional (eg, eliciting enjoyment), physical or behavioral (eg, by encouraging movement or brain activity), and social targets [15]. XR is proposed as an effective way to reduce acute pain associated with medical procedures in adults [16-18] and children through distraction [16,19,20]. With chronic pain, XR emphasizes physical and cognitive-behavioral targets [21]. Still, our understanding of the mechanisms of action and efforts to maximize the potential of these approaches for patients with either type of pain is just beginning to develop.

Evidence to date is predominantly focused on XR to reduce pain intensity in the context of acute pain broadly and specifically procedural pain [19,22-25], including the perioperative period for adult and pediatric patients. However, several recent reviews [17,19,22,25] underscore the modest impacts on pain intensity and distress due to heterogeneity of samples, small sample sizes, methodological issues, lack of digital comparators, and lack of standardization in the outcomes being measured. All these reviews and meta-analyses call for more focused, higher-quality research on the efficacy and effectiveness of XR for acute pain, establishing the best comparators and outcomes and how to tailor and scale up to individual patients and contexts.

XR as a component of a biopsychosocial pain treatment plan for chronic pain (including recurrent pains) is becoming increasingly prevalent [17]. For example, the US Food and Drug Administration recently announced the marketing of an XR system to help reduce pain in patients aged ≥18 years with chronic low back pain at home [26]. The growing affordability and portability of VR systems allow for potentially feasible applications of XR to various chronic pain conditions in clinical and home environments. To date, published work specifically focused on VR for chronic pain demonstrates support for using XR to augment established pain treatments [27-29], including physical activity [30], mirror therapy for complex regional pain syndrome [31], and biofeedback for headache [32]. There is growing evidence for the utility of XR to promote short-term reductions in pain intensity [17] and fear of pain as well as improved function through exposure-based paradigms in adult populations [17,33,34]. The intersection of these areas, that is, the use of XR to achieve multiple benefits (eg, reduction of pain, disability, fear, or behavioral avoidance) over a more extended period in rehabilitative interventions for patients with chronic pain, is only emerging.

### Objectives

**Figure 1:**
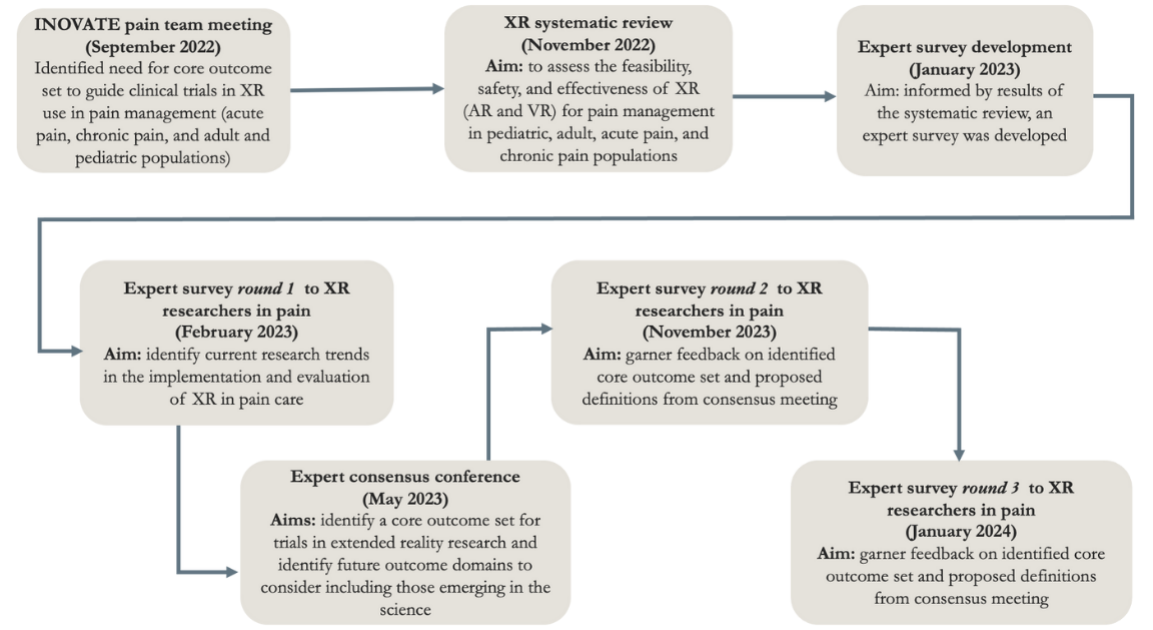
Study flow through the multiphasic approach. AR: augmented reality; INOVATE: Interdisciplinary Network on Virtual and Augmented Technologies for Pain collaborative; VR: virtual reality; XR: extended reality.

We have reached a pivotal moment in pain management where XR can potentially provide breakthroughs in our treatment of patients with both acute and chronic pain across the life span. Therefore, heeding the National Institutes of Health call for data harmonization through use of common data elements across clinical pain trials [35], and mirroring recommendations for core outcome sets (COSs) in pediatric and adult trials for acute and chronic pain more broadly [36], we need a COS to inform the design and evaluation of potential pain XR interventions. Our aim in this study was to develop a COS for both acute and chronic pain XR interventions across the life span. Following the framework and process described by Outcome Measures in Rheumatology (OMERACT), a global organization dedicated to improving care of rheumatic diseases through advancing the design and quality of clinical studies [37,38], we undertook a multiphase approach to identifying core outcomes for XR interventions for pain. The first phase involved 2 systematic reviews to determine the most used outcome domains in published research to date. In the second phase, we undertook a Delphi survey approach to collect perspectives about current treatment of acute, recurrent, and chronic pain using XR from pediatric and adult researchers and clinicians to understand clinically meaningful outcomes for XR that should be routinely measured. The inclusion of reports of ongoing (vs solely completed or published) work is particularly crucial in the field of XR intervention development given the vast discrepancies in the pace of technology innovation compared to completion and publication of clinical studies. The final phase was to reach consensus on mandatory, optional, and emerging outcome domains in XR trials for pain informed by the COS for pediatric pain trials developed by Palermo et al [36] and McGrath et al [39]. The second part of this last phase was a survey to finalize the operational definitions of the COS with exemplars (see Figure 1 for the study flow).

## Methods

### Ethical Considerations

Ethics approval was deemed unnecessary as no identifiable human data were collected, in line with institutional guidelines. However, consideration was taken to ensure anonymity and voluntary participation, and no personal identifying information was collected, stored, or reported at any stage of the survey. The expert panel survey was deemed exempt from ethics board review approval by the Boston Children’s Hospital institutional review board. Consent language was included in the instructions, and completion of the survey was considered implicit informed consent. Ethics approval was obtained from the Hospital for Sick Children for the consensus meeting. The expert panel was not compensated for their role in the panel; however, the expenses associated with travel to and accommodation during the consensus meeting were reimbursed. All participants consented to consensus meeting participation. Participant privacy was ensured through several efforts. No information about consensus meeting participants were shared without explicit permission from participants. Participants were reminded each day of the meeting that they should not share any personal identifying information from the consensus meeting outside of the meeting workgroup. Privacy of data was ensured through limiting access to study information to the researchers and SickKids Clinical Research Monitor and all data were stored in a secure, locked location.

### Study Overview

We modeled our efforts on the process by Palermo et al [36] and McGrath et al [39] of establishing a COS for pediatric chronic pain clinical trials and the original Pediatric Initiative on Methods, Measurement, and Pain Assessment in Clinical Trials (PedIMMPACT) consensus processes. We used the OMERACT framework [37,38] to choose outcome domains using clinician and researcher input followed by an expert consensus meeting that included people with lived experience. A steering committee comprised cochairs (JNS and FA) who designed the project in collaboration with key members of the Interdisciplinary Network on Virtual and Augmented Technologies for Pain collaborative (LES, DEL, Jeffrey I Gold, and Christopher King), as well as 3 trainees (BNR, CWH, and GM) and our key knowledge user (CO, a patient with lived experience). This group has broad expertise with acute and chronic pain XR and pain-focused clinical trials. JNS also has previous experience as part of the PedIMMPACT consensus group, an effort to establish core outcomes for pediatric pain clinical trials that informed our current process [39]. The steering committee met several times to develop each phase, including planning for the systematic reviews (phase 1); expert surveys (phase 2); and, finally, the consensus conference on outcome domains (phase 3), which was to be based on findings from phases 1 and 2. Following the OMERACT methodology and their 3-layer onion framework [37], the committee prioritized the domains identified in the Delphi survey as (1) the core set of domains mandatory for all trials; (2) important domains with optional inclusion; and (3) the research agenda, which is defined as domains that hold potential but require further research before inclusion in the mandatory or important layers. The onion concept was recently updated to allow for designating some mandatory outcomes that apply to specific subsets of populations.

### Systematic Reviews of XR

We conducted a systematic review (PROSPERO registration 307153) [40] of the literature to summarize studies that have examined the safety, feasibility, or effectiveness of XR as a treatment for acute, recurrent, or chronic pain in pediatric and adult populations using the PRISMA (Preferred Reporting Items for Systematic Reviews and Meta-Analyses) guidelines [41]. The primary objective was to summarize study methods and outcomes, and the secondary objective was to summarize study findings. Major electronic databases (CINAHL, Cochrane Central, Embase, MEDLINE, and PsycINFO) were searched. The search period was from inception to March 2023. The reference management software Covidence (Veritas Health Innovation) [42] was used. A total of 4 reviewers independently decided on the publications’ eligibility. To be included in the review, studies needed to (1) be conducted with participants aged 0 to 65 years with acute or chronic pain; (2) use immersive technologies (VR or AR); (3) assess XR safety, feasibility, acceptability, or effectiveness; and (4) be peer-reviewed primary literature, including observational or interventional studies. Disagreements about study selection were resolved by 2 members of the team (CWH and GM). Due to the volume of studies found for the review, it was then split into separate reviews of the pediatric and adult literatures. A data extraction form was then pilot-tested, and a team of researchers extracted article data. For the purposes of the consensus conference, data were extracted from each article and included study authors, year, country, type of pain treated, number of participants, participant ages and sex, implementation setting, study design, control group description where applicable, XR software and hardware, research procedures, and the outcomes assessed. The measures used to assess outcomes, time points of assessment, outcome results, and adverse events were also extracted. Each article was coded, and an evidence gap map was created to visualize the frequency of areas researched across populations (pediatric or adult), pain type (eg, acute wound care or chronic musculoskeletal pain), and assessed outcome domains (eg, pain intensity or daily functioning). The full details of the pediatric systematic review are described in the study by Hess et al [40], and the full details of the adult systematic review are described in a manuscript currently in preparation (Rosenbloom et al, unpublished data, March 2025).

### Delphi Expert Survey (Round 1)

Through targeting authors of papers included in the systematic reviews and via announcements to relevant listserves (pediatric pain) and professional societies (Pain in Childhood Special Interest Group and the International Association for the Study of Pain), we identified individuals with expertise in clinical use or research of XR interventions for pain management. Obtaining Delphi panel input of this type is recommended when the collective opinions of experts in a field are needed to solve a specific issue [43], when consensus-derived criteria are needed in situations of clinical uncertainty [44], or where evidence-based practice procedures have yet to be established [45]. These experts completed a survey designed to capture their current use of XR for pain and solicit opinions regarding the most important outcome domains and measures to assess in XR clinical trials.

The first-round Delphi survey included questions to elucidate who is currently delivering XR for pain (eg, clinicians or research assistants), in what settings, and with what populations (eg, age groups and pain conditions). Experts were surveyed about the types of hardware and software used and how their XR use was funded. Experts were asked about the outcomes of their XR interventions, including what domains they measured when evaluating their interventions, what constructs they assessed within those domains, and the specific measures they used to assess outcomes. Finally, they were asked to rate the perceived importance of a variety of possible domains and constructs representing potential outcomes and mechanisms of XR interventions for pain using a numeric scale from 0 to 10 (0=not at all important; 10=extremely important).

Surveys were administered through a REDCap (Research Electronic Data Capture; Vanderbilt University) interface hosted by the Boston Children’s Hospital. Experts were invited to participate in the study through an email invitation explaining the purpose of the study and providing instructions and a REDCap link to access and complete the study survey. A second round of the survey was administered following the consensus meeting and is described in the following section.

REDCap data were downloaded to SPSS (version 26; IBM Corp) for data analysis. Data were also analyzed using R statistic software (R Foundation for Statistical Computing) [46]. Data were summarized using frequencies and measures of central tendency. Data were examined based on the full sample as well as separately based on whether respondents worked in pediatric or adult care and whether they focused on acute or chronic pain. Given that several respondents reported expertise across the life span, we focused our results primarily on the full sample, with subgroup analyses presented where most informative.

### Consensus Meeting

A 2-day consensus conference was held in Toronto, Ontario (in person), to determine the minimal COS for XR clinical trials for pediatric and adult patients. The conference consisted of 21 members who were chosen because of their expertise in the development or evaluation of XR in pain treatment, as well as 2 people with lived experience. The meeting was informed by the results of the systematic review (phase 1) and Delphi survey (phase 2). A nominal group process of round-robin voting was used in the consensus conference meeting to identify the final recommendations on core outcome domains for XR research, including operational definitions for each identified domain. A clear majority (≥75%) was required to indicate consensus of the participants at each step of the nominal group process. The consensus conference was facilitated by JNS and WG, who have chaired similar meetings [47,48] and have expertise in XR as well as pediatric and adult pain, respectively. Following the consensus conference, a survey was created with the core outcomes that were voted on, including the categorization of each outcome domain (ie, mandatory, important to consider, or emerging research), as well as their developed definitions. Respondents were asked to rate the importance of each included domain and express agreement or disagreement with the proposed definition of each outcome. If respondents disagreed with the developed definition, they were asked to provide qualitative feedback to inform the refinement of the outcome definition. Ratings and feedback were then analyzed and informed an additional iteration of the survey, which was resent to all consensus members for a final vote and feedback.

### Delphi Survey Rounds 2 and 3

Following the work of the consensus meeting, the proposed domains and definitions were circulated to the same set of experts contacted for round 1 of the Delphi survey with the goal of finalizing definitions and examples for each domain. Respondents were asked to indicate agreement or disagreement with the definitions and examples derived from the consensus meeting and make any further suggestions on wording. They were also asked to indicate whether they supported including the novel emerging biomarkers, neuropsychological, and participation domains at any level in the framework. Following the round 2 survey, edits were made, and domains and definitions were again sent to the same panel of experts for final review for round 3.

## Results

### Common Outcomes Identified in the Systematic Reviews

The systematic review search yielded a total 2656 titles and abstracts. Due to the volume of studies that emerged from the search, articles that focused on pediatric and adult populations were analyzed separately. A total of 194 articles were included in data extraction (n=104, 53.6% adult; n=90, 46.4% pediatric), with 4 studies being coded as both pediatric and adult given that the study sample spanned both populations. Pain treatment outcomes were grouped according to common outcomes identified in the literature and the proposed heuristic model by Trost et al [21] and included user experience; engagement; cognitive, behavioral, and physical functioning; social functioning; emotional functioning; daily functioning; pain intensity; quality of life; health care use; safety; and feasibility. Regarding pain populations, most XR research was conducted to reduce pain intensity in the context of acute pain for pediatric patients, specifically, needle poke and procedural pain. In adult populations, chronic pain was evaluated more commonly (58/104, 55.8%); very few studies (6/90, 7%) examined the role of XR in pediatric chronic pain. Across outcome domains, pain intensity and emotional functioning were the most frequently assessed in XR trials across the life span and in acute and chronic pain conditions. The findings of the systematic review, including identified outcomes, were used to inform the development of the expert panel survey. Some terminology shifted through this process as additional information emerged through review of studies in the systematic reviews and in collaboration with the coauthors. The outcome terminology presented in each section is consistent with the terminology used in each phase of the study.

### Delphi Survey Round 1

A total of 66 experts completed the Delphi survey between November 2022 and April 2023. Most respondents were from the United States (25/66, 38%) and Canada (20/66, 30%), with Europe (9/66, 14%), Australia (8/66, 12%), and China (2/66, 3%) also represented. A total of 45% (30/66) of respondents identified professionally as scientists or professors, 27% (18/66) identified as physicians, 8% (5/66) identified as nurses or nurse practitioners, 15% (10/66) identified as physiotherapists or occupational therapists, 11% (7/66) identified as research staff, and 9% (6/66) identified as psychologists (the categories were not mutually exclusive).

Regarding contexts of XR use, 79% (52/66) of respondents reported using XR in research settings, and 61% (40/66) reported using XR clinically. The most common use setting was a hospital (30/66, 45%), followed by home use (17/66, 26%), use in an outpatient clinic (17/66, 26%), use in a rehabilitation program (14/66, 21%), and use in the emergency department (11/66, 17%). A total of 71% (47/66) of respondents reported using XR to treat chronic pain, whereas 45% (30/66) reported using XR for acute pain and 14% (9/66) reported use within experimental pain paradigms. Researchers were also asked to identify the populations in which they used XR. Some reported focus on a specific age population; 36% (24/66) of respondents reported XR use exclusively in adult populations, 35% (23/66) reported use entirely in pediatric populations, and the remaining respondents did not report a specific age focus of their XR research. Trends of XR use differed based on the patient age group of interest, with 83% (19/23) of pediatric-only users using XR in acute pain settings, including venipuncture (12/19, 63%) and wound care (9/19, 47%). Among those using XR exclusively with adult patient populations, 79% (19/24) reported using XR primarily with patients with chronic pain, with the most common presenting condition being musculoskeletal pain (8/19, 42%). A total of 14% (9/66) of the respondents reported XR use in experimental (nonclinical) settings. Table 1 shows the expert demographic and XR use context, and Table 2 shows the survey findings on target patient populations.

**Table 1:** Expert survey demographic data (N=66).

				All, n (%)		Adult (n=24), n (%)		Pediatric (n=23), n (%)
**Expert primary context**								
	Clinical			40 (61)		11 (46)		17 (74)
	Research			52 (79)		23 (96)		18 (78)
	Quality improvement			6 (9)		1 (4)		4 (17)
	Medical education			5 (8)		2 (8)		1 (4)
	Recreational			7 (11)		2 (8)		1 (4)
**Expert role**								
	Scientist or professor			30 (45)		13 (54)		7 (30)
	Physician			18 (27)		1 (4)		11 (48)
	Nurse practitioner or nurse			5 (8)		2 (8)		2 (9)
	Psychologist			6 (9)		4 (17)		0 (0)
	Research staff			7 (11)		3 (12)		4 (17)
	Physiotherapist or occupational therapist			10 (15)		6 (25)		2 (9)
	Child life specialist			3 (5)		0 (0)		1 (4)
	Educator			5 (8)		3 (12)		1 (4)
	Simulation specialist			3 (5)		1 (4)		1 (4)
	Other			2 (3)		1 (4)		1 (4)
**Expert country of practice**								
	Australia			8 (12)		4 (17)		1 (4)
	Canada			20 (30)		5 (21)		12 (52)
	China			2 (3)		0 (0)		2 (9)
	Germany			1 (2)		1 (4)		0 (0)
	Italy			2 (3)		0 (0)		1 (4)
	The Netherlands			1 (2)		1 (4)		0 (0)
	Turkey			1 (2)		1 (4)		0 (0)
	United States			25 (38)		8 (33)		7 (30)
	United Kingdom			5 (8)		4 (17)		0 (0)
**Context of XR^a^ use**								
	**Clinical**							
		Hospital	30 (45)		6 (25)		17 (74)	
		Community based	7 (11)		5 (21)		1 (4)	
		Outpatient	17 (26)		7 (29)		5 (22)	
		Perioperative	5 (8)		0 (0)		3 (13)	
		Emergency department	11 (17)		0 (0)		9 (39)	
		Inpatient setting	6 (9)		1 (4)		3 (13)	
		Rehabilitation	14 (21)		8 (33)		3 (13)	
		At-home use	17 (26)		11 (46)		4 (17)	
		Other	4 (6)		2 (8)		1 (4)	
	**Academic**							
		Behavioral assessment room	2 (3)		2 (8)		0 (0)	
		Research laboratory	20 (30)		12 (50)		5 (22)	
		Other	2 (3)		1 (4)		0 (0)	

^a^XR: extended reality.

**Table 2:** Delphi survey results of current pain populations being treated using extended reality (N=66).

		All respondents, n (%)	Adults, n (%)	Pediatric, n (%)
**Acute pain**		66 (100)	24 (36)	23 (35)
	IV^a^ access or venipuncture	17 (26)	1 (4)^b^	12 (52)^c^
	Port access	6 (9)	0 (0)^b^	4 (17)^c^
	Lumbar puncture	3 (5)	0 (0)^b^	1 (4)^c^
	Wound care or dressing change	14 (21)	0 (0)^b^	9 (39)^c^
	Burn care	9 (14)	0 (0)^b^	6 (26)^c^
	Perioperative pain	8 (12)	1 (4)^b^	4 (17)^c^
	Dental procedures	4 (6)	0 (0)^b^	2 (9)^c^
	Labor pain	2 (3)	1 (4)^b^	1 (4)^c^
	Minor procedure	9 (14)	1 (4)^b^	5 (22)^c^
	Vaso-occlusive crisis	4 (6)	2 (8)^b^	2 (9)^c^
	Other	2 (3)	0 (0)^b^	1 (4)^c^
**Chronic pain (primary or secondary)**		58 (88)	38 (66)^d^	20 (34)^d^
	Neuropathic pain	8 (14)^d^	2 (5)^e^	3 (15)^f^
	Complex regional pain syndrome	7 (12)^d^	2 (5)^e^	1 (5)^f^
	MSK^g^ pain	13 (22)^d^	8 (21)^e^	1 (5)^f^
	Headaches	6 (10)^d^	3 (8)^e^	1 (5)^f^
	Abdominal pain	7 (12)^d^	2 (5)^e^	3 (15)^f^
	Cancer pain	10 (17)^d^	3 (8)^e^	2 (10)^f^
	Arthritic pain	7 (12)^d^	6 (16)^e^	0 (0)^f^
	Phantom limb pain	4 (7)^d^	2 (5)^e^	1 (5)^f^
	Chronic widespread pain or fibromyalgia	8 (14)^d^	3 (8)^e^	1 (5)^f^
	Other	2 (3)^d^	2 (5)^e^	0 (0)^f^
Experimental		9 (14)	7 (78)^h^	1 (11)^h^

^a^IV: intravenous.

^b^n=24.

^c^n=23.

^d^n=58.

^e^n=38.

^f^n=20.

^g^MSK: musculoskeletal.

^h^n=9.

Regarding research design and assessment, overall, randomized controlled trials (30/66, 45%) and acceptability or feasibility studies (29/66, 44%) were the most common designs reported across the full sample of respondents, and this trend was true across studies of XR with both pediatric and adult populations. Of those who responded (47/66, 71%), 83% (39/47) reported measuring outcomes in their studies, and the most common outcomes were pain intensity (39/39, 100%), distraction (36/39, 92%), anxiety (32/39, 82%), pain-related fear (23/39, 59%), and patient satisfaction (23/39, 59%). In adult studies, the most common pain outcomes were reported to be pain intensity (26/34, 76%), distraction (18/34, 53%), and anxiety (17/34, 50%). In pediatric populations, the most common outcomes were reported to be distraction (26/33, 79%), anxiety (24/33, 73%), and pain intensity (23/33, 70%). Adverse events related to XR use were also commonly assessed across age groups, with respondents stating that they specifically assessed for dizziness (27/39, 69%), nausea or vomiting (23/39, 59%), headache (17/39, 44%), and eye strain (11/39, 28%).

When asked about the importance of specific XR outcomes, overall, there was moderate agreement on the domains of greatest importance, with mean importance ratings of >8/10 for safety or side effects (mean 8.7, SD 1.82; range 1-10); feasibility, usability, and acceptability (mean 8.8, SD 1.57; range 3-10); pain interference (mean 8.4, SD 2.31; range 0-10); pain intensity (mean 8.5, SD 1.66; range 2-10); emotional functioning, specifically fear or anxiety (mean 8.4, SD 1.90; range 0-10); and user experience or immersion (mean 8.2, SD 2.40; range 0-10). When separated into pediatric and adult researchers, those using XR with adults rated pain interference and function as most important (mean 9.0, SD 1.47; range 5-10), followed by pain intensity (mean 8.8, SD 1.19; range 7-10) and feasibility, usability, and acceptability (mean 8.8, SD 1.41; range 6-10). Among pediatric researchers, safety or side effects were rated as most important (mean 8.9, SD 1.21; range 7-10), followed by feasibility, usability, and acceptability (mean 8.8, SD 1.21; range 7-10) and emotional functioning, specifically fear or anxiety (mean 8.6, SD 1.01; range 7-10). Table 3 shows the importance ratings across all outcome domains from round 1 of the survey.

**Table 3:** Importance ratings of outcome domains identified in round 1 of the expert surveya.

	Total (n=66), mean (SD; range)	Adult (n=24), mean (SD; range)	Pediatric (n=23), mean (SD; range)
Pain intensity and quality	8.5 (1.66; 2-10)	8.8 (1.19; 7-10)	8.4 (1.22; 6-10)
Pain interference and function	8.4 (2.31; 0-10)	9.0 (1.47; 5-10)	8.4 (1.03; 7-10)
Anxiety or fear	8.4 (1.90; 0-10)	7.5 (2.88; 0-10)	8.6 (1.01; 7-10)
Physical performance	6.8 (2.66; 0-10)	7.2 (1.91; 0-10)	7.0 (2.40; 2-10)
Physiology	6.3 (2.68; 0-10)	6.0 (2.83; 0-10)	6.9 (2.21; 2-10)
User experience	8.2 (2.40; 0-10)	7.9 (2.63; 2-10)	8.4 (1.39; 6-10)
Side effects and safety	8.7 (1.82; 1-10)	8.5 (2.47; 1-10)	8.9 (1.21; 7-10)
Feasibility, usability, and acceptability	8.8 (1.57; 3-10)	8.8 (1.41; 6-10)	8.8 (1.21; 7-10)

^a^Means, SDs, and ranges were calculated using the greatest number of responses available, and the n value for each domain varies.

### Consensus Meeting

In May 2023, once the expert panel survey was completed, an in-person consensus conference was held with the steering committee and invited international experts, including 2 people with lived experience, with the goal of reaching consensus on a set of recommended outcome domains for pediatric and adult acute and chronic pain clinical XR trials. The meeting agenda included discussions about the goals of the project; earlier work conducted by the Initiative on Methods, Measurement, and Pain Assessment in Clinical Trials as well as PedIMMPACT and other more recent COSs [36,39,49,50]; and presentations of the data from the systematic reviews (BNR and CWH), expert survey results (DEL), and methodology to consider for future measure selection (future work).

**Figure 2:**
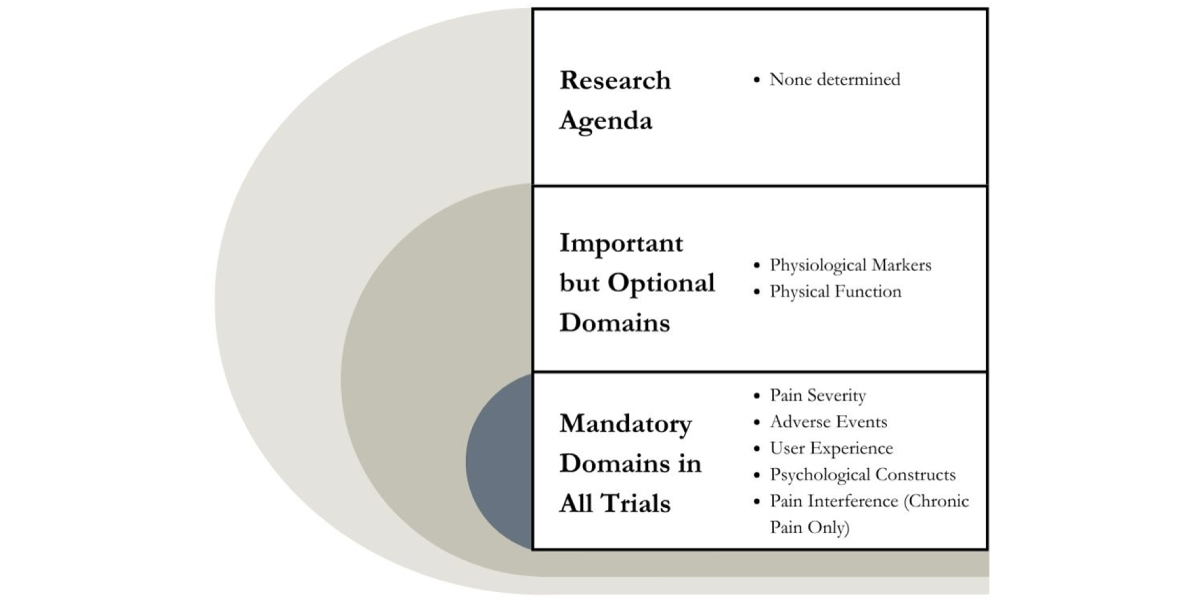
Outcome Measures in Rheumatology framework for core outcomes in extended reality research.

During the meeting, it was determined through consensus vote (>75%) that pain severity, adverse events, user experience, and psychological constructs would be considered mandatory domains to be assessed in all XR clinical trials for acute and chronic pain; for trials of chronic pain only, pain interference should also be considered a mandatory outcome. The consensus attendees had extensive discussions regarding psychological constructs as a mandatory domain. It was decided that a psychological shift when using the technology was core to capture and that the domain would be separated into cognitive and affective components. Attendees also discussed the overlap between the satisfaction and participant experience and engagement domains. It was decided that both domains would be combined into user experience as a core domain. In addition, there was substantial discussion regarding the overlap and distinctions between physiological markers, physical function, pain interference, and the construct of participation, which represented clear differences in language used across distinct professions. The occupational therapists present at the consensus conference described the construct of participation as the extent to which one can engage in their daily life demands and preferred activities, whereas others in the meeting described this phenomenon as pain interference. Ultimately, it was decided that the included construct would be pain interference as the people with lived experience in the consensus conference felt that this better represented their experiences as they perceived pain to be interfering with their ability to engage in their daily demands and preferred activities. Another outcome of this discussion was a vote regarding the inclusion of physical functioning and physiological markers, which were ultimately classified through voting as important-to-consider but optional domains as there are areas of pain XR research in which these 2 domains might be less relevant. Finally, novel biomarkers were considered an emerging domain as there is a lack of research and evidence to rise to the level of necessary to measure. It was agreed that the incorporation of biomarkers should be revisited as XR technology develops. One of the consensus meeting members did provide an informative overview of advances in technology, including biomarkers within XR. This prompted group discussion regarding the potential role of VR in the measurement of omics, eye tracking, and behavioral data. It was unanimously agreed by the group that these metrics may be important outcomes or mechanisms worthy of study that will become more central as the technology further develops. Slight adjustments to labels for outcome domains and definitions were made immediately after the meeting. Resulting outcomes for each domain are presented in Figure 2. Proposed definitions for each of the outcome domains are shown in Multimedia Appendix 1, and final importance ratings are shown in Table 4.

**Table 4:** Final importance ratings across all core outcome domains (range 1-10).

			Ratings, mean (SD; range)
**Core domains**			
	Pain severity	9.72 (0.67; 8-10)	
	Adverse events	9.39 (0.85; 8-10)	
	User experience	8.67 (0.97; 7-10)	
	Psychological constructs	8.55 (1.46; 5-10)	
	Pain interference (chronic pain only)	8.83 (1.42; 6-10)	
**Important to consider domains**			
	Physiological markers	7.62 (1.65; 4-10)	
	Physical function	7.41 (2.03; 4-10)	
**Removed domains^a^**			
	Neuropsychological	6.18 (1.87; 4-10)	
	Novel, emerging biomarkers	4.73 (2.44; 2-10)	
	Participation	6.29 (2.73; 2-10)	

^a^Social: unanimous vote to remove in round 1 (n=17).

### Delphi Survey Rounds 2 and 3

As shown in Multimedia Appendix 1, results of the second-round Delphi survey largely supported the consensus meeting outcomes. No further changes were recommended to the definitions of the outcome domains of pain severity, adverse events, or user experience. Under the core domains, minor suggestions were provided for psychological constructs, particularly for the concept of motivation and whether it should be included (within the examples vs the definition), and the definition of pain interference was edited slightly for readability. Under important-to-consider domains, no changes were suggested for physical function or physiological markers. None of the other constructs that were raised for potential inclusion (novel emerging biomarkers, neuropsychological, and participation domains) received sufficient support to justify including them in the final framework.

## Discussion

### Principal Findings

The study objectives were to reach consensus on a COS for pediatric and adult pain XR interventions using an accepted framework and methodology. In our first phase of work, briefly presented in this report, we conducted a systematic review of the current literature on XR for adult and pediatric pain to understand outcomes measured in published studies. Our review of the literature underscored that, to date, there is far more evidence of the use of XR interventions for acute and procedural pain than for chronic pain, particularly in the pediatric population. In the smaller set of published XR studies for chronic pain, most focused on adult populations. Therefore, studies of XR interventions, including feasibility trials, for pediatric chronic pain conditions are urgently needed. Our systematic review process highlighted user experience, engagement, psychological constructs or emotional functioning, physical functioning, social functioning, quality of life, pain intensity, health care use, safety, and feasibility as common outcomes assessed in pain XR studies, with pain intensity and psychological constructs being the most frequently assessed outcomes. A full report of our systematic review findings is forthcoming.

As XR innovation outpaces the rate at which clinical studies are completed and published, we did not want to rely solely on published studies to gauge the current trends in XR pain interventions. Therefore, we conducted a survey of experts *currently* using XR for pain in research or clinical contexts to understand current targets of these interventions and what specific measures are being used to assess outcomes. The results highlight that pain intensity, anxiety or fear (a specific aspect of emotional functioning), and patient satisfaction are commonly targeted outcomes of XR interventions for pain. Although distraction is commonly measured in studies of XR for pain, we conceptualized this domain as a mechanism of XR’s effects rather than an outcome. Other outcome domains supported to a lesser extent by the survey results included physiological markers (eg, heart rate variability and respiratory rate), physical functioning, and adverse events. When asked to rate the importance of specific outcome domains, experts rated pain intensity, pain interference (for chronic pain), psychological or emotional functioning, satisfaction, immersion, and adverse events as most important, in descending order. However, there was a lack of consensus regarding specific measures used to capture these core outcomes, with a wide range of measures currently in use.

On the basis of the systematic review–informed expert panel and subsequent expert survey, we conclude that established outcomes commonly used in pain XR research include assessments of pain intensity, psychological or emotional functioning (either at a general level or specific aspects such as fear of pain), functional impairment or pain interference, user experience and satisfaction, immersion or engagement, and safety and adverse events. Convergence on these outcomes across our first 2 steps in our COS process strongly suggested that these were important domains for further consideration.

In a 2-day consensus meeting with a range of stakeholders including people with lived experience, our steering committee reached consensus on a COS layered within the OMERACT framework that includes 4 mandatory domains to be assessed in all clinical trials of pain XR interventions: pain severity, user experience, adverse events, and psychological constructs. For trials focused on chronic pain, an additional mandatory core outcome of pain interference was included. The committee also identified 2 optional domains important for inclusion in some trials of pain XR interventions depending on the goals of the study: physiological markers and physical functioning. This consensus meeting was followed by a second and third round of the Delphi survey, which largely provided further support for the framework generated in the consensus meeting, with minor alterations for clarity and readability of the definitions and examples of core and important-to-consider constructs.

This multistage consensus-building process elucidated some important topics that did not ultimately end up in our framework on key outcomes for pain XR research. As discussed in our consensus meeting, novel biomarkers have begun to be used in other areas of VR research. This has included the use of eye tracking to assess pupil reactivity to improve safety in aeronautics [51], the use of VR testing to assess executive dysfunction in patients with multiple sclerosis [52] and Parkinson disease [53] as well as those diagnosed with obsessive-compulsive disorder [54], and the use of VR in experimental paradigms for understanding anxiety development [55]. Similarly, neuropsychological indicators of attention, memory, and other constructs may become useful areas of measurement. Although the group determined that it is premature to incorporate these into the framework at this stage in the field’s development, we anticipate further growth in these areas, potentially leading to future framework expansion.

Overall, our recommendations are well aligned with recent recommendations for a COS for chronic pain trials in pediatrics more broadly (ie, not focused on XR), as well as the National Institute of Mental Health’s Research Domain Criteria initiative, a framework created to guide mental health research to improve diagnosis, prevention, intervention, and cures [56]. Recent published recommendations include 8 core domains: pain intensity, physical functioning, symptoms and adverse events, global satisfaction with treatment, emotional functioning, role functioning, sleep, and economic factors. Areas in which our recommendations diverge from this set focus on aspects that are specific and important to digital technology interventions. For example, user experience is a domain that is uniquely important to capture in research on XR-based interventions as aspects such as immersiveness and presence are particular strengths specific to this emerging technology. One drawback of the PedIMMPACT guidance is that it can be burdensome to assess 8 outcome domains. Participants, especially people with lived experience, in our consensus conference emphasized the need to limit burden on patients participating in outcome studies. In addition, it may be challenging to find valid assessment tools within each of these domains. Our group sought to strike a balance between thoroughness and feasibility in recommended approaches to demonstrating the effectiveness of XR interventions for pain. In designating mandatory domains, we strove to facilitate comparison of a minimum set of outcomes across trials to ultimately develop an evidence base that allows patients and health care providers to choose a treatment based on known beneficial effects and potential risks.

There are other movements in evidence-based medicine with which this COS could also align, including individualizing treatments for patients and shared decision-making with health care providers. Consistent use of this COS across acute and chronic pain XR interventions will produce evidence on benefits and risks of each treatment along a set of common outcome domains. Dissemination of this evidence may then arm patients with knowledge to make informed decisions with their health care providers based on assessment of risk and benefit profiles and their own individual preferences. Patients may be able to individualize evaluation of treatment benefits (ie, tying benefit of treatment to what actually matters to each individual patient) by choosing treatments that have known benefit in domains that are of the most individual importance to them (eg, choosing a treatment that targets fear of pain over a treatment aimed at increasing range of motion).

We also highlight that the COS is considered appropriate for a range of study types, including feasibility studies, clinical trials, and longitudinal clinical outcomes. Our survey with health care providers suggested that there was no differentiation of outcomes for the purpose of a clinical trial versus a longitudinal registry. Thus, similar to what has already been conducted with PedIMMPACT recommendations, we encourage those studying longitudinal clinical outcomes in clinical databases and registries to follow the same guidance.

Our findings should be interpreted considering several limitations. First, we recruited convenience samples for the Delphi survey. Given the high percentage of North American participants, the results may not accurately reflect global perspectives. In addition, respondents may not reflect the full range of health care providers using XR with patients with pain given our recruitment through specific professional societies; for example, child life and rehabilitation specialists were likely underrepresented in these societies and likely offer important perspective on uses of XR for pain. Additional surveys to solicit the views of people with lived experience on important outcome domains of XR interventions would have strengthened our overall process. Of note, we did incorporate people with lived experience in our consensus meeting. However, the findings should also be interpreted through the lens of the group of individuals who were part of the consensus conference. Although efforts were made to diversify the consensus meeting group, members were predominantly White individuals, and most held positions within academic medical centers or higher education. Members were diverse with regard to their professional identity (researchers, psychologists, physicians, occupational therapists, and nurses), and during discussions, the impact of professional training background was important, resulted in disagreements regarding language, and impacted the individual perceived importance of each domain. The primary population that each participant worked with also impacted their perception of outcome importance. For example, individuals working predominantly with patients with chronic pain emphasized the importance of physical functioning over pain intensity, whereas those in the acute pain setting emphasized more the importance of pain intensity. It is also likely that group members were impacted by their own research in this domain and the outcome domains that they had identified in past or current research studies, which may have impacted their rating of various outcome domains. An additional limitation is that, although the OMERACT framework was generally identified as relevant, there were domains generated in the rheumatology consensus-building process that were not appropriate for our purposes (eg, “death” as a core outcome). There are also domains relevant to XR outcome assessment that are specific to XR, such as immersiveness of the XR experience. Finally, as noted, our terminology describing some domains shifted in the course of our multiphase process to reach a final set of core outcomes. This is appropriate because it reflected what we learned along the way but does introduce some variability across phases.

Future iterations of the COS are expected and encouraged to advance its utility. Future work may specifically address some of the sampling limitations in our study (eg, broader range of health care providers and more balanced international representation) or may focus on refining the domains (evaluating applicability across acute and chronic pain contexts and across the patient life span). Our committee’s next steps include identifying relevant validated outcome measures and reaching consensus on recommendations for measures for each of the identified domains. On the basis of our expert survey, which showed a wide range of measures used within the commonly measured domains and low consensus on which measures should serve as gold standards, we anticipate this next step to be a challenging one. Nonetheless, this is an important goal to achieve to develop standard approaches for evaluating the effectiveness of pain XR interventions and guiding the research process within this fast-growing area of inquiry.

### Conclusions

In conclusion, XR is an exciting and promising digital health tool whose applications for reducing pain are just emerging. To realize the promise of this realm of innovation more rapidly and promote consistency in the growing evidence base, this report describes the initial steps in a process of identifying a COS for use in evaluating the effects of XR in pain treatment. The identification and adoption of a uniform set of outcome domains and measures will promote standardized evaluation and easy, widespread access to the latest opportunities, tools, and resources. Addressing these gaps in XR research will help reduce pain and improve quality of life for children, youth, and adults with acute, recurrent, and chronic pain.

## Appendix

Multimedia Appendix 1 Core domains and definitions for extended reality trials for pain.

## Abbreviations

AR: augmented reality
COS: core outcome set
OMERACT: Outcome Measures in Rheumatology
PedIMMPACT: Pediatric Initiative on Methods, Measurement, and Pain Assessment in Clinical Trials
PRISMA: Preferred Reporting Items for Systematic Reviews and Meta-Analyses
REDCap: Research Electronic Data Capture
VR: virtual reality
XR: extended reality

## Data Availability

The datasets generated or analyzed during this study are available from the corresponding author on reasonable request.
