# Supplementary material for: Developing a Core Outcome Set for Pediatric and Adult Acute and Chronic Pain Extended Reality Trials: Delphi Consensus-Building Process
Source: J Med Internet Res. 2025 May 23;27:e58947. doi: 10.2196/58947 (PMC12144474; doi:10.2196/58947)
Supplement: Multimedia Appendix 1 [file jmir_v27i1e58947_app1.docx]

**Table S1.** Core Domains and Definitions for XR trials in pain

| **CORE DOMAINS** | | | |
| --- | --- | --- | --- |
| **DOMAIN** | **Original Definition** | **Round 1 Definition** | **Final Definition** |
| Pain Severity | Perception of the severity of pain including how intense pain is and how frequently it occurs (Palermo et al., 2021). Need to have at least one measure of pain severity that should match the population under study.  **Descriptors/Examples:**  **Acute**   - e.g., quality, duration, intensity, unpleasantness   **Chronic**   - e.g., quality, episode duration, intensity, frequency, unpleasantness | Perception of how intense pain is and how frequently it occurs. Need to have at least one measure of pain severity that should match the population under study.  **Descriptors/Examples:**  Examples can include intensity, duration, quality, frequency and unpleasantness (for chronic pain only) | Perception of how intense pain is and how frequently it occurs. Need to have at least one measure of pain severity that should match the population under study.  **Descriptors/Examples:**  Examples can include intensity, duration, quality, frequency and unpleasantness (for chronic pain only) |
| Adverse Events | Any untoward occurrence or unfavorable and unintended sign, symptom or disease that impacts the health or well-being of a research participant who is administered an investigational product or any other research procedure(s), and which does not necessarily have a causal relationship with the product.  **Descriptors/Examples:**  Within this, need to define for the specific study  **Related/unrelated**  e.g., event as a result of the XR or unrelated to the XR  **Anticipated/unanticipated**  e.g., dizziness, nausea, nausea/simulator sickness  **Severity**  e.g., mild, moderate, severe | Any untoward occurrence or unfavorable and unintended sign, symptom or disease that impacts the health or well-being of a research participant who is administered an XR product.  **Descriptors/Examples:**  **Related/unrelated**  e.g., event as a result of the XR or unrelated to the XR  **Anticipated/unanticipated**  e.g., simulator sickness (dizziness, nausea), headache, unsteadiness, visual disturbance  **Severity of signs and symptoms**  e.g., mild, moderate, severe | Any untoward occurrence or unfavorable and unintended sign, symptom or disease that impacts the health or well-being of a research participant who is administered an XR product.  **Descriptors/Examples:**  **Related/unrelated**  e.g., event as a result of the XR or unrelated to the XR  **Anticipated/unanticipated**  e.g., simulator sickness (dizziness, nausea), headache, unsteadiness, visual disturbance  **Severity of signs and symptoms**  e.g., mild, moderate, severe |
| User Experience | Subjective user experience related to the technical aspects of the XR system  **Descriptors/Examples:**  **Immersion:**can refer both to the subjective user assessment (i.e., a sense of being "caught up and absorbed" in the virtual world) and to the XR system configuration (e.g., a 3-dimensional 360-degree virtual environment presented through HMD vs a 2-dimensional presentation on a computer screen).  **Embodiment:** "sense of having one's body" and reflects the integration of multiple sensory signals (e.g., visual, tactile, and kinesthetic), which in turn can be manipulated by XR  **Presence:**subjective experience of being in one place or environment, even when one is physically situated in another  **Engagement:** Reported or observed use of XR technology (e.g., frequency, duration, % fidelity to prescribed use)  **Enjoyment:** reported enjoyment or positive experience using the XR intervention | User experience (subjective, proxy, observer reported) related to the technical aspects of the XR system (software/hardware)  **Descriptors/Examples:**  **Immersion:**can refer both to the subjective user assessment (i.e., a sense of being "caught up and absorbed" in the virtual world) and to the XR system configuration (e.g., a 3-dimensional 360-degree virtual environment presented through HMD vs a 2-dimensional presentation on a computer screen).  **Embodiment:** "sense of having one's body" and reflects the integration of multiple sensory signals (e.g., visual, tactile, and kinesthetic), which in turn can be manipulated by XR  **Presence:**subjective experience of being in one place or environment, even when one is physically situated in another  **Engagement:** Reported or observed use of XR technology (e.g., frequency, duration, % fidelity to prescribed use),ease of use  **Enjoyment:** reported enjoyment or positive experience using the XR intervention | User experience (subjective, proxy, observer reported) related to the technical aspects of the XR system (software/hardware)  **Descriptors/Examples:**  **Immersion:**can refer both to the subjective user assessment (i.e., a sense of being "caught up and absorbed" in the virtual world) and to the XR system configuration (e.g., a 3-dimensional 360-degree virtual environment presented through HMD vs a 2-dimensional presentation on a computer screen).  **Embodiment:** "sense of having one's body" and reflects the integration of multiple sensory signals (e.g., visual, tactile, and kinesthetic), which in turn can be manipulated by XR  **Presence:**subjective experience of being in one place or environment, even when one is physically situated in another  **Engagement:** Reported or observed use of XR technology (e.g., frequency, duration, % fidelity to prescribed use), ease of use  **Enjoyment:** reported enjoyment or positive experience using the XR intervention |
| Psychological Constructs | Individuals' cognitive or emotional responses to intervention delivered or changes in cognitive or emotional processes following engagement with the XR.  **Descriptors/Examples:**  **Emotion**: Relating to one's expression, perception, and conceptualization of emotions/feelings   - e.g., depression, mood, anxiety, well-being, affect   **Cognition**: Relating to one's thoughts, attitudes, beliefs, and desires   - e.g., pain-related fear, catastrophizing, self-efficacy, pain acceptance | Individuals' cognitive, affective, or motivational responses on engagement with the XR  **Descriptors/Examples:**  **Emotion**: Relating to one's expression, perception, and conceptualization of emotions/feelings   - e.g., depression, mood, anxiety, well-being, affect   **Cognition**: Relating to one's thoughts, attitudes, beliefs, and desires   - e.g., pain-related fear, worry, self-efficacy | Individuals' emotional and cognitive engagement with the XR  **Descriptors/Examples:**  **Emotion**: Relating to one's expression, perception, and experience of emotions/feelings   - e.g., sadness, awe, fear, motivation   **Cognition**: Relating to one's thoughts, attitudes, beliefs, and desires   - e.g., pain-related fear, worry, self-efficacy, motivation   **Depressive/Anxiety Symptoms** |
| Pain Interference (Chronic Pain Only) | How much pain interferes with engagement in social, physical, and recreational activities (Palermo et al., 2021).  **Descriptor/Example:**  This can include specifics of type of interference (quality, frequency of engagement).   - e.g., exercise, sport, play, social exchange, work, education, or home. | How much pain gets in the way of engagement in social, physical, and recreational activities.  **Descriptor/Example:**  This can include specifics of type of interference (quality, frequency of engagement).   - e.g., exercise, sport, play, social exchange, work, education, or home. | The extent to which pain interferes an individual's engagement in social, physical, and recreational activities.  **Descriptor/Example:**  This can include specifics of type of interference (quality, frequency of engagement).   - e.g., activities of daily living, exercise, sport, recreation/leisure, social, work, education, or home |
| **IMPORTANT TO CONSIDER DOMAINS** | | | |
| **DOMAIN** | **Original Definition** | **Round 1 Definition** | **Final Definition** |
| Physiological Markers | Markers of both stress and pain  **Descriptors/Examples:**   - Cardiovascular System (e.g., HRV, Pulse, HR, BP) - Autonomic System (e.g., Skin Conductance, Cortisol) - Visual System (e.g., Eye Tracking) - Central Nervous System (e.g., EEG, Brain Imaging) - Respiratory (e.g., O2 Saturation) | A measurable and quantifiable biological parameter that serves as an indicator of a particular physiological state during the use of XR products.  **Descriptors/Examples:**   - Cardiovascular System (e.g., HRV, Pulse, HR, BP) - Autonomic System (e.g., Skin Conductance, Cortisol) - Visual System (e.g., Eye Tracking) - Central Nervous System (e.g., EEG, Brain Imaging) - Respiratory (e.g., O2 Saturation, respirator rate [bpm]) | A measurable and quantifiable biological parameter that serves as an indicator of a particular physiological state during the use of XR products.  **Descriptors/Examples:**   - Cardiovascular System (e.g., HRV, Pulse, HR, BP) - Autonomic System (e.g., Skin Conductance, Cortisol) - Visual System (e.g., Eye Tracking) - Central Nervous System (e.g., EEG, Brain Imaging) - Respiratory (e.g., O2 Saturation, respirator rate [bpm]) |
| Physical Function | Targeting improved engagement in movement/kinematic patterns, physical activities, and/or exercise  **Descriptors/Examples:**   - Sensor-based motion-tracking of movement, performance-based measures (timed up and go, 6 minute walk) - Self-reported physical function (e.g., SF-36 physical functioning subscale [general measure] or a condition-specific measure) - Subjective/objective measures of physical activity (e.g., international physical activity questionnaire [IPAQ], accelerometry) | Targeting i) movement/kinematic patterns; ii) engagement in movement, physical activities, or exercise; and/or iii) capacity to undertake activities of daily living and physical function  **Descriptors/Examples:**   - Sensor-based motion-tracking of movement (Garmin, Fitbit, Apple watch), performance-based measures (timed up and go, 6 minute walk) - Self-reported physical function (e.g., SF-36 physical functioning subscale [general measure] or a condition-specific measure) - Subjective/objective measures of physical activity (e.g., international physical activity questionnaire [IPAQ], accelerometry) | Physical function is a multi-dimensional concept, relating to i) physical body functions and structures (or impairments in), ii) physical activities (or limitations in), iii) physical participation or involvement of people in all areas of life (or restrictions within these)  **Descriptors/Examples:**   - Self-report measures of physical body functions/structures, physical activities, or physical participation in life activities - Performance-based measures of physical body functions/structures or physical activities, including: - Physical body functions/structures (impairments): Movement/kinetic patterns (e.g., sensory-based motion tracking of movement), strength, range of motion, balance, etc. - Physical activities (limitations): 6-minute walk test, timed up and go, 9 hold peg test, Timed functional and arm shoulder test, wearables. |
| **REMOVED DOMAINS** | | | |
| **DOMAIN** | **Original Definition** | **Round 1 Definition** | **Final Definition** |
| Novel emerging biomarkers | Provide objective indicators of physiological, cognitive, and emotional status  **Examples:**   - Omics - Basic Animal Research - Big Data - Machine Learning - Eye-gaze tracking | Biological markers of a biological state which provides indicators of physiological, cognitive, affective, and motivational status  **Examples:**   - Omics - Pre-clinical Research | Domain Removed |
| Neuropsychological | The main domain of neuropsychological encompasses various processes, each with its specific subconstructs that further define and explore these neuropsychological functions  **Examples:**attention, perception, memory, language, cognitive control, and working | Various cognitive and behavioral functions having to do with the brain and the central nervous system processes  **Examples:**attention, perception, memory, language, cognitive control, and working memory | Domain Removed |
| Participation | **Definition:**The ability to engage in personally meaningful activities. Meaningful activities are congruent with an individual's value system, help to meet individual needs, often allow the individual to potentially/eventually demonstrate competence and mastery, and have value within that individual's social and cultural group. These activities provide purpose, control and agency. They can also provide a sense of belonging to the community. This outcome also signifies the return to "normal" life as defined by the individual  **Examples:**Individual's perceived ability to take part in specific work, study, family, travel, hobbies, recreational, social, civic, and religious/spiritual activities as well as social relationships in a way that results in satisfactory performance and outcomes for that individual. Global Perceptions that one is achieving engagement in meaningful activities. | Domain Removed | Removed |
| Social | Unanimous vote to remove (n=17) | Domain Removed | Removed |
